# Scalp cooling with adjuvant/neoadjuvant chemotherapy for breast cancer and the risk of scalp metastases: systematic review and meta-analysis

**DOI:** 10.1007/s10549-017-4185-9

**Published:** 2017-03-08

**Authors:** Hope S. Rugo, Susan A. Melin, Jeff Voigt

**Affiliations:** 1 University of California San Francisco Helen Diller Family Comprehensive Cancer Center, UCSF Medical Center at Mount Zion, 1600 Divisadero St, San Francisco, CA 94115 USA; 2 Wake Forest University Baptist Medical Center, Medical Center Blvd, Fl 3, Winston Salem, NC 27157 USA; 3 Medical Device Consultants Ridgewood, LLC, 99 Glenwood Rd, Ridgewood, NJ 07450 USA

**Keywords:** Scalp metastasis, Scalp cooling, Breast cancer

## Abstract

**Purpose:**

The risk of scalp metastases in patients using scalp cooling for preservation of hair during chemotherapy has been a concern but is poorly described.

**Methods:**

A systematic review and meta-analysis of longitudinal studies was undertaken to evaluate the effect of scalp cooling versus no scalp cooling on the risk of scalp metastasis in patients treated for breast cancer with chemotherapy. Electronic databases, journal specific, and hand searches of articles identified were searched. Patients were matched based on disease, treatment, lack of metastatic disease, and sex.

**Results:**

A total of 24 full-text articles were identified for review. Of these articles, ten quantified the incidence of scalp metastasis with scalp cooling over time. For scalp cooling, 1959 patients were evaluated over an estimated mean time frame of 43.1 months. For no scalp cooling, 1238 patients were evaluated over an estimated mean time frame of 87.4 months. The incidence rate of scalp metastasis in the scalp cooling group versus the no scalp cooling group was 0.61% (95% CI 0.32–1.1%) versus 0.41% (95% CI 0.13–0.94%); *P* = 0.43.

**Conclusion:**

The incidence of scalp metastases was low regardless of scalp cooling. This analysis suggests that scalp cooling does not increase the incidence of scalp metastases.

**Electronic supplementary material:**

The online version of this article (doi:10.1007/s10549-017-4185-9) contains supplementary material, which is available to authorized users.

## Introduction

Breast cancer is a common malignancy in the western world; it is estimated that more than 246,660 women in the USA will be diagnosed in 2016 with this disease [1, 2]. Most of these women are treated surgically with curative intent, although neoadjuvant chemotherapy may be given first to improve surgical options. Adjuvant therapy including hormonal and chemotherapy, as well as local radiation therapy, is a commonly used modality following definitive surgery to reduce the risk of local and systemic recurrence. Adjuvant chemotherapy has been shown to delay or prevent recurrence in early-stage breast cancer, and recent studies as well as ongoing work are helping to define the group of patients who are most likely to benefit from this treatment [3, 4]. A cancer diagnosis is psychologically distressing, and decisions about adjuvant therapy compound this distress. Alopecia is a disturbing side effect of almost all effective adjuvant chemotherapy regimens. Chemotherapy is associated with a number of toxicities, but alopecia is the most publicly visible sign of this treatment with psychological impact [5–8]. Scalp cooling to prevent chemotherapy-induced alopecia has been in use since the 1970s, and was recently cleared for marketing in the United States [9]. Existing and emerging data have demonstrated excellent or good prevention of alopecia caused by commonly used chemotherapeutic regimens [10]. The protection from alopecia offered by scalp cooling is thought to be due to both vasoconstriction resulting in reduced blood flow in the scalp, and reduced metabolic rate in the hair follicles [6].

The primary concern that has limited the use of scalp cooling devices in the United States is the possibility that scalp cooling could increase the risk for scalp metastases [10]. Scalp metastases are a rare site of metastatic disease in breast cancer [11, 12].

A recent review of the literature on scalp metastasis following adjuvant chemotherapy for early-stage breast cancer [13] found it unlikely that the incidence of scalp metastasis might increase after scalp cooling. However, this study reviewed the incidence of both skin and scalp metastasis with and without adjuvant chemotherapy in patients with early- and late-stage breast cancer as well as other cancers, and with highly variable follow-up, which included patients on whom the follow-up time was not identified.

The intent of this systematic review and meta-analysis is to examine the effect of scalp cooling versus no scalp cooling on the incidence of scalp metastasis in patients being treating for breast cancer with adjuvant chemotherapy with identified follow-up.

## Methods

A systematic review of the literature was undertaken using the following search terms: chemotherapy AND breast cancer AND scalp metastas*.

The following electronic databases were searched:

- PubMed (searched on October 13, 2016 & February 15, 2017)
- Google (searched on October 13, 2106 & February 15, 2017; first 4 pages of hits)

The following online journals were searched:

- Breast Cancer Research and Treatment (searched on October 13, 2016 & February 15, 2017)
- Supportive Care in Cancer (searched on October 13, 2016 & February 15, 2017)
- European Journal Oncology Nursing (searched on October 13, 2016 & February 15, 2017)
- European Journal Cancer (searched on October 13, 2016 & February 15, 2017)
- Journal Clinical Oncology (searched on October 13, 2016 & February15, 2017).

Hand searches of all articles included in the analysis were undertaken on October 14–15, 2016 & February 16–17, 2017.

Two authors independently reviewed each of the studies and used a data collection form to determine inclusion eligibility (see Appendix 1). A third author acted as arbiter in those situations where disagreement existed between the first two authors.

### Inclusion criteria used in the qualitative and quantitative (meta-analysis) review

Articles which evaluated patients treated for breast cancer with chemotherapy and, with and without the use of scalp cooling technology and, examined the longer-term sequelae of this therapy (with identified follow-up timeframes) including scalp metastasis were included in this analysis.

### Exclusion criteria used

Patients who had other types of early-stage cancers, studies that did not examine longer-term sequelae (i.e., only reported on the immediate short term results of the cancer treatment), and studies including patients who were not treated with chemotherapy were excluded from the analysis.

### Statistical analysis

MedCalc statistical software (Version 16.8.4) was used to calculate the incidence rates and confidence intervals of the combined studies with and without the use of scalp cooling over time. A *P* value was then calculated to determine if there were a statistically meaningful difference between the two rates. A weighted (weighted based on the number of patients in each trial) average of the follow-up durations was then calculated along with standard deviations.

## Results

**Fig. 1:**
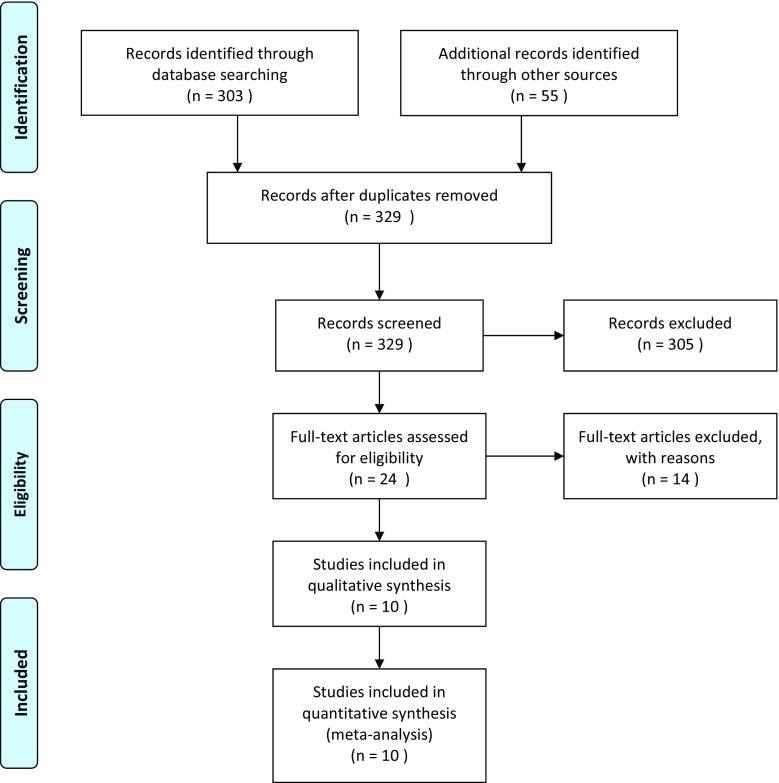
PRISMA 2009 flow diagram

Figure 1 shows the identification, screening, eligibility, and inclusion as part of the systematic review. A total of 24 full-text studies were identified as eligible for review.

### Included studies

Ten studies which reported on scalp metastasis were included in the analysis, of which five reported on scalp cooling only [13–17], four reported on scalp cooling versus no scalp cooling [11, 18–20], and one on no scalp cooling [12] (Table 1).

**Table 1:** Included Studies

Study	Scalp cooling		No scalp cooling		Length of follow-up (months) scalp cooling median	Weighted length of follow-up scalp cooling	Length of follow-up (months) no scalp cooling median	Weighted length of follow-up no scalp cooling	Characteristics
	Scalp mets	Total pts	Scalp mets	Total pats					
Lemieux et al. [11]	6	553	1	87	69	19.478	64	4.498	First time breast cancer patients. Study undertaken in Canada. Mainly T1 and T2 tumor size; stage 1 & 2, treated with mainly cyclophosphamides and doxorubicin
Parker [14]	0	6			12	0.037			Stage 4 recurrent disease. Treated with IV CMF (2 cycles)
Protiere et al. [18]	0	77	0	109	44	1.729			First time breast cancer patients. 4 cycles of adjuvant IV chemotherapy with mitoxantrone + cyclophosphamide. Antiemetics also administered. Study undertaken in France
Ridderheim [15]	0	3			15	0.023			Adjuvant treatment breast cancer
Ron et al. [19]	0	19	0	16	14	0.136	14	0.181	Breast cancer patients treated with cyclophosphamide, methotrexate, and 5- fluorouracil [CMF]; unclear as to stage of breast cancer
Rugo [17]	0	101			29.5	1.521			Early-stage breast cancer patients
Spaëth et al. [20]	3	770	0	141	36	14.150	36	4.100	93% breast cancer patients. Treated with IV chemo mainly anthracyclines and/or taxotere. Unclear as to stage of breast cancer
Tollenaar et al. [35]	0	35			46	0.822			Patients treated with cyclophosphamide + doxorubicin + 5-fluorouracil on first operative day (one course of treatment). Unclear as to stage of cancer
van de Sande [12]			4	885			110	78.635	Stage 4 + lymph nodes
van den Hurk et al. [13]	3	395			26	5.242			treated with CMF; unclear as to stage of breast cancer
Totals	12	1959	5	1238					
Averages					32.39	43.14	56	87.41	

### Excluded studies

Fourteen studies were excluded for the following reasons: No follow-up or unclear follow-up time following completion of chemotherapy (total of 10) [21–30]; patients used scalp cooling for treatment of metastatic disease (total of 2) [31, 32]; treatment for breast cancer was not specified (one study) [33]; and one study was excluded due to unclear cancer type at presentation [34] (Table 2).

**Table 2:** Studies excluded with reasons

Study	Reason for exclusion
Christodoulou [21]	Zero out of 30 breast cancer patients developed a scalp metastasis. However, there was no mention of the follow-up time in this patient cohort. Scalp cooling was employed
Campos-Gomez [23]	Sixty-eight patients in scalp cooling were reported as followed up on, but it was unclear as to the timeframe of follow-up. No mention as well of scalp metastasis
Christodoulou [22]	Two out of 227 breast cancer patients developed scalp metastasis. However, there was no mention of the follow-up time in this patient cohort. Scalp cooling was employed
Dean [24]	Fifty-eight breast cancer patients treated with doxorubicin and with scalp cooling were reported on for scalp metastasis, but there was no mention of the follow-up time
Johansen [31]	One patient in scalp cooling group who already had metastatic breast cancer (in the liver) experienced scalp metastasis (*n* = 61); all patients in group already had metastatic primary or recurrent cancer originating from the breast
Kargar [34]	Unclear as to type of primary cancer patients had
Lemenager [32]	Breast cancer patients treated with chemotherapy (*n* = 88); all had metastatic disease and were treated with scalp hypothermia. No mention of scalp metastasis
Lookingbill [33]	No mention of how patients with breast cancer were treated; There were 18 patients with scalp metastases out of 707 primary breast cancer patients. (18/707 = 2.5%). There was also no mention of the follow-up time as to when scalp metastases occurred
Massey [25]	Breast cancer patients treated with chemotherapy (*n* = 94) treated with Paxman cooling system. None of these patients developed scalp metastases during the study period. Unclear as to the length of follow-up on these patients
Middleton et al. [26]	Twenty-four patients with breast cancer receiving adjuvant chemotherapy. No length of follow-up noted. No scalp metastases noted
Nangia et al. [30]	No long-term follow-up on patients in randomized controlled trial
Peck et al. [27]	One patient in scalp cooling group subsequently presented with scalp metastasis. However, patient already had widespread metastatic cancer (*n* = 10); unclear as to follow-up
Satterwhite [28]	One patient in scalp cooling group already had scalp metastasis (*n* = 12); no follow-up
van den Hurk [29]	Breast cancer patients treated with chemotherapy in the Dutch scalp cooling registry (*n* = 1216). No patients developed scalp metastasis during the study period of 2006–2009. However, unclear as to the length of follow-up on these patients

### Effect of scalp cooling vs non-scalp cooling on the outcome of scalp metastasis

There were 12 cases of scalp metastases out of 1959 patients where scalp cooling was employed (Table 1). The incidence rate was 0.61% (95% CI 0.32–1.1%). These patients were followed for an estimated mean of 43.14 months (weighted mean average). There were 5 cases of scalp metastasis out of 1238 patients where scalp cooling was not used (Table 1). The incidence rate was 0.4% (95% CI 0.13–0.9%). These patients were followed for an estimated mean of 87.4 months (weighted mean average). There was no statistically meaningful difference between the two comparison groups (with and without scalp cooling) (*P* = 0.43).

## Discussion

The possibility that scalp cooling protects the scalp from the beneficial effects of adjuvant chemotherapy has been a concern that has limited the use of these devices in the United States. However, this systematic review and meta-analysis examining patients with breast cancer receiving chemotherapy while using scalp cooling for hair preservation does not support this concern and, demonstrates no statistical difference in the incidence of scalp metastasis between patients using scalp cooling vs. no scalp cooling. This analysis further complements the van den Hurk 2013 study [13] (which also found no statistically meaningful difference) and adds additional longer-term study data (with confirmed duration of follow-up of breast cancer specifically to identify scalp metastasis) to further substantiate the low incidence of scalp metastases [15, 18, 35].

Scalp metastases occur rarely in breast cancer (with metastases more commonly occurring in other areas of skin including chest wall [36]) and, as reviewed above, scalp metastases seem to accompany and usually occur following the diagnosis of widespread metastatic disease. Interestingly, in a sensitivity analysis which added back those studies that were excluded due to no mention of follow-up time and/or type of cancer treatment but where breast cancer was identified as the primary source, scalp cooling and no scalp cooling were identified, no other metastatic cancer was present, and scalp metastases were identified [22, 24–26, 29, 33]; a statistically significant difference in the incidence of scalp cooling between scalp cooling and no scalp cooling was found [11–20] (scalp cooling incidence 0.4%; 95% CI 0.21–0.66% vs. no scalp cooling: 1.2%; 95% CI 0.75–1.8%; *P* = 0.006).

### Additional questions considered

Does adjuvant chemotherapy reduce the risk for scalp metastases in breast cancer patients? The ability of adjuvant chemotherapy to specifically reduce scalp metastases presumes that there are dormant micrometastatic cells already seeded in the scalp from the primary tumor at the time of diagnosis of early-stage disease. However, it is much more likely that adjuvant chemotherapy effects occult metastatic cells in other sites that might eventually metastasize to the scalp [37], as this site (scalp) of metastatic disease is uncommon.

Does scalp cooling increase the incidence of scalp metastases as the first sign of recurrent breast cancer? Scalp metastases are very rarely reported as the first site of metastatic recurrence in patients with early-stage breast cancer. The National Surgical Adjuvant Breast and Bowel Project (NSABP) reported in a communication to Judith Dean [38] that two patients in a sample of 7800 women had metastases to scalp as the first site of recurrent disease. One of these patients had received adjuvant chemotherapy. The incidence of scalp metastases as the first site of recurrence can therefore be estimated to be around 0.025% (2/7800).

Is it possible that scalp cooling used in conjunction with adjuvant chemotherapy increases the risk for scalp metastases as the site for first recurrence? Based on available data, this appears to be highly unlikely. Two cases of scalp metastases as the first detected metastatic site in patients with breast cancer previously treated with adjuvant chemotherapy together with scalp cooling were described by Lemieux [39]. The first patient presented with a scalp metastasis as the first site of metastatic disease 9 years following breast cancer chemotherapy, but had only used scalp cooling during 2 of 4 cycles of doxorubicin and cyclophosphamide. Many other sites of metastases were found in this patient a few months later. The second patient used scalp cooling in only one out of 6 cycles of adjuvant cyclophosphamide, methotrexate, and 5-fluorouracil, and then was treated for a local recurrence 5 years later with surgery and 6 cycles of epirubicin 100 mg/m^2^ without scalp cooling. It is highly unlikely that scalp cooling used in one out of 12 chemotherapy cycles in this patient at high risk for recurrent disease contributed to the finding of a scalp metastasis 7 years after her initial diagnosis. In both cases, it is quite unlikely that there is any association of scalp cooling with subsequent development of a scalp metastasis.

Considering the exceedingly low incidence of scalp metastasis as a first site of recurrence (or in general), the risk appears small for scalp cooling to increase the incidence of scalp metastases in patients with breast cancer. In addition, the concept that scalp cooling could increase the incidence of metastases to the scalp suggests dormant cells in the scalp are responsible for recurrence—again unlikely. Based on what is now understood about the biology of breast cancer, and the low incidence of scalp metastases as the site of first recurrence, it is very unlikely that scalp cooling contributes to the risk of metastatic recurrence.

### Study limitations

The analysis undertaken was retrospective in nature. As well, most studies did not examine scalp metastasis as a primary endpoint. These types of studies have inherent biases.

The scalp cooling comparison arm (*n* = 1,959) evaluated patients over an estimated weighted mean of 42.1 months versus an estimated weighted mean of 87.4 months for non-scalped-cooled patients. The assumption in calculating these weighted mean averages was that the distributions from which the sample means came from were relatively normal (bell-shaped and symmetric). Further, since the sample sizes of several of the studies evaluated were large [7, 11–13, 17, 18] (comprising over 98% of the patients evaluated), the median and mean were assumed to be fairly close in value. Based on these factors, we believe the weighted mean value is a reasonable approximation of the follow-up timeframes. Follow-up on scalp cooling patients over a longer period of time is ongoing [17, 30].

## Conclusion

Based on this extensive review and meta-analysis, scalp cooling is highly unlikely to increase the incidence of scalp metastases in patients with early-stage breast cancer receiving adjuvant chemotherapy. Van den Hurk 2013 [13] stated: “We found it rather unlikely that the incidence of scalp metastases might increase at all after scalp cooling and; that a very small proportion of patients receiving chemotherapy (with or without scalp cooling) are at risk for developing metastases at this site.” Based on this analysis, we would concur with van den Hurk.

## Electronic supplementary material

Below is the link to the electronic supplementary material.

Supplementary material 1 (DOCX 23 kb)

## References

[1] Howlander N, Noone AM, Krapcho M, et al (eds). SEER cancer statistics review, 1975–2012, National Cancer Institute, Bethesda. http://seer.cancer.gov/csr/1975_2012/ based on November 2014 SEER data submission, posted to the SEER web site, April 2015

[2] American Cancer Society. Cancer facts & figures 2016. American Cancer Society, Atlanta. http://www.cancer.org/research/cancerfactsstatistics/cancerfactsfigures2016/

[3] National Comprehensive Cancer Network (NCCN) NCCN clinical practice guidelines in oncology. Breast Cancer. Version 1.2016. 2015 Nov 18; http://www.nccn.org/professionals/physician_gls/pdf/breast.pdf

[4] Henry NL, Somerfield MR, Abramson VG (2016). Role of patient and disease factors in adjuvant systemic therapy decision making for early-stage, operable breast cancer: American society of clinical oncology endorsement of cancer care Ontario guideline recommendations. J Clin Oncol.

[5] Choi EK, Kim I-R, Chang O (2014). Impact of chemotherapy-induced alopecia distress on body image, psychosocial well-being, and depression in breast cancer patients. Psycho-Oncology.

[6] Rosman S (2004). Cancer and stigma: experience of patients with chemotherapy-induced alopecia. Patient Educ Couns.

[7] Lemieux J, Maunsell E, Provencher L (2008). Chemotherapy-induced alopecia and effects on quality of life among women with breast cancer: a literature review. Psycho-Oncology.

[8] Mols F, van den Hurk C, Vingerhoets AJJM (2009). Scalp cooling to prevent chemotherapy-induced hair loss: practical and clinical considerations. Support Care Cancer.

[9] FDA Medical Devices (2016). General and plastic surgery devices; classification of the scalp cooling system to reduce the likelihood of chemotherapy-induced alopecia. Food and Drug Administration, HHS. Fed Regist.

[10] Grevelman EG, Breed WPM (2005). Prevention of chemotherapy-induced hair loss by scalp cooling. Ann Oncol.

[11] Lemieux J, Amireault C, Provencher L, Maunsell E (2009). Incidence of scalp metastases in breast cancer: a retrospective cohort study in women who were offered scalp cooling. Breast Cancer Res Treat.

[12] van de Sande MA, van den Hurk CJ, Nreed WP (2010). [Allow scalp cooling during adjuvant chemotherapy in patients with breast cancer; scalp metastases rarely occur. Ned Tijdschr Geneeskd.

[13] van den Hurk CJG, van de Poll-Franse Breed WPM (2013). Scalp cooling to prevent alopecia after chemotherapy can be considered safe in patients with breast cancer. The Breast.

[14] Parker R (1987). The effectiveness of scalp hypothermia in preventing cyclophosphamide-induced alopecia. Oncol Nurs Forum.

[15] Ridderheim M, Bjurberg M, Gustavsson A (2003). Scalp hypothermia to prevent chemotherapy-induced alopecia is effective and safe: a pilot study of a new digitized scalp-cooling system used in 74 patients. Support Cancer Care.

[16] Tollenar RAEM, Liefers GJ, Repelaer van Driel OJ (1994). Scalp cooling has no place in the prevention of alopecia in adjuvant chemotherapy for breast cancer. Eur J Cancer.

[17] Rugo HR, Klein P, Melin SA (2017). Association between use of a scalp cooling device and alopecia after chemotherapy for breast cancer. JAMA.

[18] Protière C, Evans K, Camerio J (2002). Efficacy and tolerance of a scalp-cooling system for prevention of hair loss and the experience of breast cancer patients treated by adjuvant chemotherapy. Support Care Cancer.

[19] Ron LG, Kalmus Y, Kalmus Z (1997). Scalp cooling in the prevention of alopecia in patients receiving depilating chemotherapy. Support Care Cancer.

[20] Spaëth D, Luporsi E, Weber B (2008). Efficacy and safety of cooling helmets (CH) for the prevention of chemotherapy-induced alopecia (CIA): a prospective study of 911 patients (pts). J Clin Oncol.

[21] Christodoulou C, Klouvas G, Efstathiou E (2002). Effectiveness of the MSC cold cap system in the prevention of chemotherapy-induced alopecia. Oncology.

[22] Christodoulou C, Tsalakos G, Galani E (2006). Scalp metastases and scalp cooling for chemotherapy-induced alopecia prevention. Ann Oncol.

[23] Campos-Gomez S, Cruz LR, Morales NC (2015). Safety and efficacy of scalp hypothermia to prevent chemotherapy-induced alopecia: a study of a scalp-cooling system used in breast cancer women in a Mexican public hospital. J Clin Oncol.

[24] Dean JC, Griffith KS, Cetas TC (1983). Scalp hypothermia: a comparison of ice packs and the Kold Kap^®^ in the prevention of doxorubicin-induced alopecia. J Clin Oncol.

[25] Massey CS (2004). A multicentre study to determine the efficacy and patient acceptability of the Paxman scalp cooler to prevent hair loss in patients receiving chemotherapy. Eur J Oncol Nurs.

[26] Middleton J, Franks D, Buchanan RB (1985). Failure of scalp hypothermia to prevent hair loss when cyclophosphamide is added to doxorubicin and vincristine. Cancer Treat Rep.

[27] Peck HJ, Mitchell H, Stewart AL (2000). Evaluating the efficacy of scalp cooling using the Penguin cold cap system to reduce alopecia in patients undergoing chemotherapy for breast cancer. Eur J Oncol Nurs.

[28] Satterwhite B, Zimm S (1984). The use of scalp hypothermia in the prevention of doxorubicin-induced hair loss. Cancer.

[29] van den Hurk CJG, Peerbooms M, van de Poll-Franse LV (2012). Scalp cooling for hair preservation and associated characteristics in 1411 chemotherapy patients—results of the Dutch Scalp Cooling Registry. Acta Oncol.

[30] Nangia J, Wang T, Osborne C (2017). Effect of scalp cooling device on alopecia in women undergoing chemotherapy for breast cancer. The SCALP randomized clinical trial. JAMA.

[31] Johansen LV (1985). Scalp hypothermia in the prevention of chemotherapy-induced alopecia. Acta Radiol.

[32] Lemenager M, Lecomte S, Bonneterre ME (1997). Effectiveness of cold cap in the prevention of docetaxel-induced alopecia. Eur J Cancer.

[33] Lookingbill DP, Spangler N, Helm KF (1993). Cutaneous metastases in patients with metastatic carcinoma: a retrospective study of 4020 patients. J Am Acad Dermatol.

[34] Kargar M, Sarvestani RS, Khojastech JN, Heidari MT (2011). Efficacy of penguin cap as scalp cooling system for intervention of alopecia in patient undergoing chemotherapy. J Adv Nurs.

[35] Tollenar RAEM, Liefers GJ, Repelaer van Driel OJ (1994). Scalp cooling has no place in the prevention of alopecia in adjuvant chemotherapy for breast cancer. Eur J Cancer.

[36] Krathen RA, Orengo IF, Rosen T (2003). Cutaneous metastasis: a meta-analysis of data. South Med J.

[37] National Institute for Health and Care Excellence. Early and locally advanced breast cancer: diagnosis and treatment. Clinical Guideline. Published: 25 February 2009. Accessed on 18 Jan 2017 : nice.org.uk/guidance/cg80

[38] Dean JC, Griffith KS, Cetas TC (1983). Scalp hypothermia: a comparison of ice packs and the Kold Kap in the prevention of doxorubicin-induced alopecia. J Clin Oncol.

[39] Lemieux J, Desbiens C, Hogue JC (2011). Breast cancer scalp metastasis as first metastatic site after scalp cooling: two cases of occurrence after 7- and 9-year follow up. Breast Cancer Res Treat.

