# Supplementary material for: Scalp cooling with adjuvant/neoadjuvant chemotherapy for breast cancer and the risk of scalp metastases: systematic review and meta-analysis
Source: Breast Cancer Res Treat. 2017 Mar 8;163(2):199–205. doi: 10.1007/s10549-017-4185-9 (PMC5410200; doi:10.1007/s10549-017-4185-9)
Supplement: Supplementary file 1 — Supplementary material 1 (DOCX 23 kb) [file 10549_2017_4185_MOESM1_ESM.docx]

**Appendix 1: Data extraction form:**

**Name of person/reviewer extracting data:**

**Author of article:**

**Title:**

**Source (e.g. Journal title):**

**Date of study:**

**Study location (geographical):**

**Care setting (e.g. hospital/outpatient setting):**

**Inclusion/exclusion criteria (list of patient inclusion and exclusion criteria)**

***Inclusion*:**

***Exclusion*:**

**Sample size:**

*number in each arm of trial*

**Patient baseline characteristics:**

- *age range:*
- *gender:*
- *medical condition(s):*
- *treated with chemotherapy* **YES NO**

**TRIAL DESIGN DETAILS:**

**Longitudinal study YES NO**

**Included scalp metastasis as an endpoint YES NO**

**Intervention details**

- *care setting:*
- *treatment group(s):*
- *control(s):*

*co-interventions:*

*duration of intervention:*

**Outcome measures**

- *what were they?*
- *methods of assessing outcome measures:*
- *length of follow-up (mths):*

**Analysis:**

- *description of analysis employed:*
- *statistical methods:*
- *comparisons made:*
- *subgroups considered:*

**Results:**

*Missing data:*

*length of follow-up:*

*reasons for withdrawal:*

lost to follow-up:

Number of adverse events:

*Intervention arm (1):*

*Control arm (2):*
